# Correction: The large-scale organization of shape processing in the ventral and dorsal pathways

**DOI:** 10.7554/eLife.34464

**Published:** 2017-12-20

**Authors:** Erez Freud, Jody C Culham, David C Plaut, Marlene Behrmann

Freud E, Culham JC, Plaut DC, Behrmann M. 2017. The large-scale organization of shape processing in the ventral and dorsal pathways. *eLife* **6**:e27576. doi: 10.7554/eLife.27576.

Published 5, October 2017

In Figure 2C, for the dorsal right pathway, the first component and the second component were mistakenly switched. The data described in the text is correct, and this is a graphic error that does not affect the reported results or conclusions.

We have corrected the error by replacing Figure 2C with the correct one.

The corrected figure is shown here:

**Figure 1:**
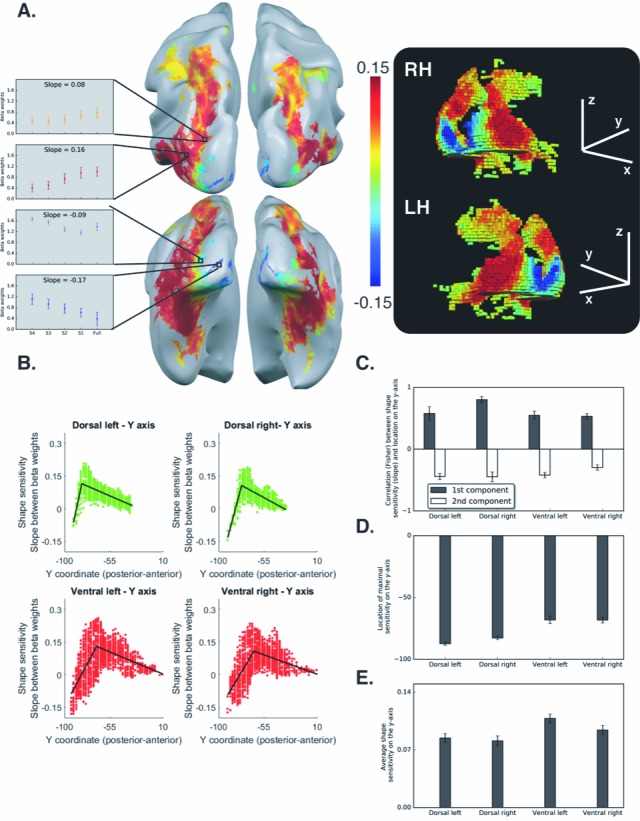


The originally published Figure 2 is also shown for reference:

**Figure 2:**
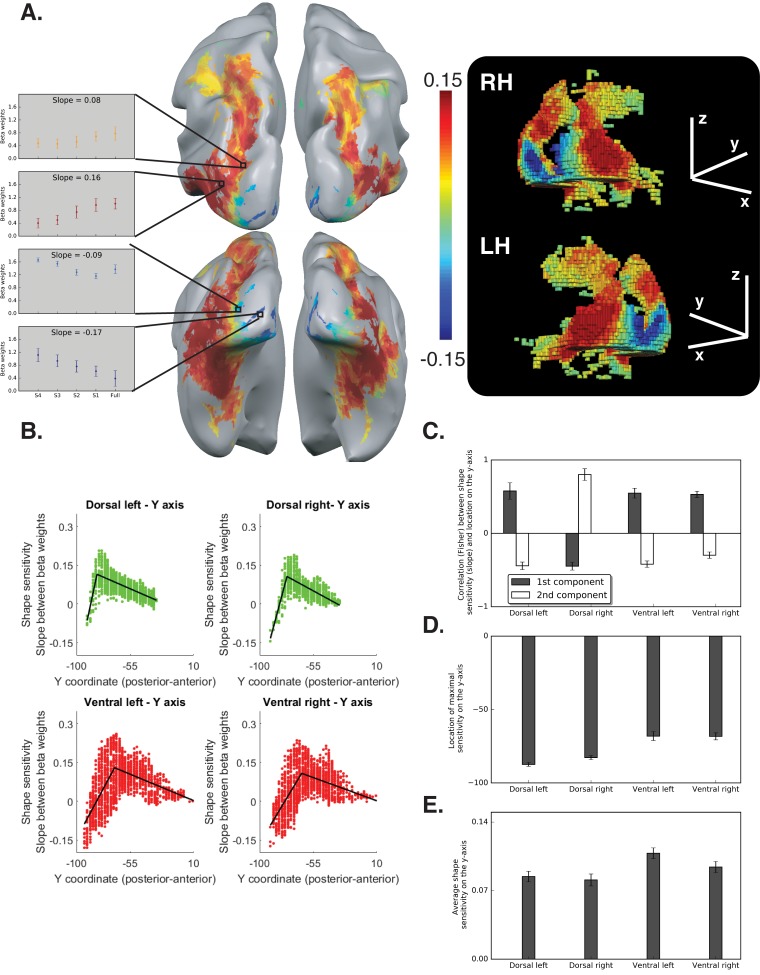


The article has been corrected accordingly.

