# Supplementary material for: The large-scale organization of shape processing in the ventral and dorsal pathways
Source: eLife. 2017 Oct 5;6:e27576. doi: 10.7554/eLife.27576 (PMC5659821; doi:10.7554/eLife.27576)
Supplement: Source code 1. [file elife-27576-code1.docx]

%This code was used for the voxelwise analysis (Freud, Culham, Plaut and Behrmann). It calls text files created by BV and utilizes the SLM toolbox. 8/3/2017.For any questions please write to erezfreud@gmail.com

%voxel data from BV. The code correlates and calculate reg. coff between different

 %mni cord (y/z) and the linear slope.

clear all;

clear all;

path = 'define the path to main folder';

no_cat_flag=0 % enables to analyze the data based on pictures of object and tools (averaged) (0) or based on picture of objects (excluding all tools) (1)

Experiment 1 subjects:

%subjects={'160224104136','160224122535','160301110546','160307093730','160307111751','160307130827','160310120847','160420131627','160421091747','160427140455','160510164658'}; %

Experiment 2 subjects

subjects={'512_exp2','024_exp2','036_exp2','314_exp2','351_exp2','408_exp2','355_exp2','236_exp2','622_exp2','100_exp2','133_exp2'}; % exp2

roi = {'ventral_r','ventral_l','dorsal_r','dorsal_l'}; %don’t change the order of rois

x=1:5;

% The first loop defines all the visually responsive voxels. It calls a txt file created in BV that includes that beta weights and the t value of all voxels that were included in the initial mask (based on a group-averaged RFX analysis).

group_voxels=cell(397456,9,length(roi));

for r=1:4

    A = importdata([path 'group\voxel analysis\' roi{r} '_exp1and2_MNI_revision.txt'],' ',24);

    temp=A.data;

    temp1=sortrows(temp, [4,6,8,10]);

    temp_final=num2cell(temp1);

    group_t=temp_final(:,[1 2 3 4 6 8 10 12]);

    clear temp_final

    clear temp1

    clear temp

    clear A

    for v=2:length(group_t)

    if cell2mat(group_t(v-1,6))==cell2mat(group_t(v,6)) %reduce resampled voxels

    group_voxels(v,4:9,r)=num2cell(NaN);

    group_voxels(v,1:3,r)=group_t(v,1:3);

    else

        if r>2

            if ((cell2mat(group_t(v,4))>2.6 || cell2mat(group_t(v,5))>2.6  || cell2mat(group_t(v,6))>2.6 || cell2mat(group_t(v,7))>2.6)  || cell2mat(group_t(v,8))>2.6)

            group_voxels(v,1:8,r)=group_t(v,:);

            group_voxels(v,9,r)=num2cell(v);

            else

            group_voxels(v,4:9,r)=num2cell(NaN);

            group_voxels(v,1:3,r)=group_t(v,1:3);

            end

        else

            if (cell2mat(group_t(v,4))>2.6 || cell2mat(group_t(v,5))>2.6  || cell2mat(group_t(v,6))>2.6  || cell2mat(group_t(v,7))>2.6)  || cell2mat(group_t(v,8))>2.6

            group_voxels(v,1:8,r)=group_t(v,:);

            group_voxels(v,9,r)=num2cell(v);

            else

            group_voxels(v,4:9,r)=num2cell(NaN);

            group_voxels(v,1:3,r)=group_t(v,1:3);

            end

        end

    end

            group_voxels(1,4:9,r)=num2cell(NaN);

            group_voxels(1,1:3,r)=group_t(v,1:3);

    end

end

 ventral_right_voxels=sortrows( cell2mat(group_voxels(:,:,1)),[9,1 -2 3]);

 ventral_left_voxels=sortrows( cell2mat(group_voxels(:,:,2)),[9,-1,-2,3]);

 dorsal_right_voxels=sortrows( cell2mat(group_voxels(:,:,3)),[9,1,-2,3]);

 dorsal_left_voxels=sortrows( cell2mat(group_voxels(:,:,4)),[9,-1,-2,3]);

  clear group_voxels group_t

 ventral_right= nan(sum(~isnan(ventral_right_voxels(:,9))),9,length(subjects));

 ventral_left=nan(sum(~isnan(ventral_left_voxels(:,9))),9,length(subjects));

 dorsal_right=nan(sum(~isnan(dorsal_right_voxels(:,9))),9,length(subjects));

 dorsal_left=nan(sum(~isnan(dorsal_left_voxels(:,9))),9,length(subjects));

% The result of the first loop is four matrices (ventral right, ventral left, dorsal right and dorsal left) each of them includes all the voxels that were found to be visually responsive (and their coordinates). The txt files were created in BV (voxel-wise GLM analysis)

% The second loop computes the slope (shape sensitivity) for each participant and each voxel.

for s= 1:length(subjects) % all subjects

    for r=1:length(roi) % all rois

    if no_cat_flag==1

    A = importdata([path subjects{s} '\voxel analysis\' roi{r} '_mni_revision.txt'],' ',24);

    [path subjects{s} '\voxel analysis\' roi{r} '_mni_revision.txt']

    else

    A = importdata([path subjects{s} '\voxel analysis\' roi{r} '_mni_revision_onlyobjects.txt'],' ',24);

    end

    temp=A.data;

    if r==1

        for v=1:length(ventral_right)

        temp_intersect=intersect(intersect(find((temp(:,1))==ventral_right_voxels(v,1)),find((temp(:,2))==ventral_right_voxels(v,2))),find((temp(:,3))==ventral_right_voxels(v,3))); % detects the voxel

        ventral_right(v,1:8,s)=(temp(temp_intersect,[1 2 3 4 6 8 10 12])); % imports the coordinates (1,2,3) and the beta weights 4-intact 6-s4 8-s16 10 s-64 12-s256

        slopeO=polyfit(x,ventral_right(v,4:8,s),1); % computes the linear fit of the beta weights

        ventral_right(v,9,s)=slopeO(1); %objects

        end

    elseif r==2

       for v=1:length(ventral_left)

        temp_intersect=intersect(intersect(find((temp(:,1))==ventral_left_voxels(v,1)),find((temp(:,2))==ventral_left_voxels(v,2))),find((temp(:,3))==ventral_left_voxels(v,3)));

        ventral_left(v,1:8,s)=(temp(temp_intersect,[1 2 3 4 6 8 10 12]));

        slopeO=polyfit(x,ventral_left(v,4:8,s),1);

        ventral_left(v,9,s)=slopeO(1);

       end

    elseif r==3

       for v=1:length(dorsal_right)

        temp_intersect=intersect(intersect(find((temp(:,1))==dorsal_right_voxels(v,1)),find((temp(:,2))==dorsal_right_voxels(v,2))),find((temp(:,3))==dorsal_right_voxels(v,3)));

        dorsal_right(v,1:8,s)=(temp(temp_intersect,[1 2 3 4 6 8 10 12]));

        slopeO=polyfit(x,dorsal_right(v,4:8,s),1);

        dorsal_right(v,9,s)=slopeO(1);

       end

    else

        for v=1:length(dorsal_left)

        temp_intersect=intersect(intersect(find((temp(:,1))==dorsal_left_voxels(v,1)),find((temp(:,2))==dorsal_left_voxels(v,2))),find((temp(:,3))==dorsal_left_voxels(v,3)));

        dorsal_left(v,1:8,s)=(temp(temp_intersect,[1 2 3 4 6 8 10 12]));

        slopeO=polyfit(x,dorsal_left(v,4:8,s),1);

        dorsal_left(v,9,s)=slopeO(1);

        end

    end

    end

             a=s

end

%reverse slopes (negative to positive, such that positive slope equals to greater beta weights for the less distorted images

ventral_right_avg(:,9)=-ventral_right_avg(:,9);

ventral_left_avg(:,9)=-ventral_left_avg(:,9);

dorsal_right_avg(:,9)=-dorsal_right_avg(:,9);

dorsal_left_avg(:,9)=-dorsal_left_avg(:,9);

%reverse slopes (negative to positive)

ventral_right(:,9,:)=-ventral_right(:,9,:);

ventral_left(:,9,:)=-ventral_left(:,9,:);

dorsal_right(:,9,:)=-dorsal_right(:,9,:);

dorsal_left(:,9,:)=-dorsal_left(:,9,:);

 %

 %This loop measures the distance from posterior- inferior voxel based on the z and y coordinates

for i=1:length(ventral_left)

    xy=[min(ventral_left(:,2,1)),max(ventral_left(:,3,1));ventral_left(i,2,1),ventral_left(i,3,1)];

    ventral_left(i,10,:)=pdist(xy,'euclidean');

end

ventral_left_avg(:,10)=ventral_left(:,10,1)

for i=1:length(ventral_right)

    xy=[min(ventral_right(:,2,1)),max(ventral_right(:,3,1));ventral_right(i,2,1),ventral_right(i,3,1)];

    ventral_right(i,10,:)=pdist(xy,'euclidean');

end

ventral_right_avg(:,10)=ventral_right(:,10,1)

for i=1:length(dorsal_right)

    xy=[min(dorsal_right(:,2,1)),min(dorsal_right(:,3,1));dorsal_right(i,2,1),dorsal_right(i,3,1)];

    dorsal_right(i,10,:)=pdist(xy,'euclidean');

end

dorsal_right_avg(:,10)=dorsal_right(:,10,1)

for i=1:length(dorsal_left)

    xy=[min(dorsal_left(:,2,1)),min(dorsal_left(:,3,1));dorsal_left(i,2,1),dorsal_left(i,3,1)];

    dorsal_left(i,10,:)=pdist(xy,'euclidean');

end

dorsal_left_avg(:,10)=dorsal_left(:,10,1)

%%

%%%%

%This part of the code utilizes the SLM toolbox to computes the piecewise regression for each participant. The function slm_optimize (see at the bottom of this code) is used to optimize the R^2^. The SLM toolbox is an external toolbox that needs to be downloaded.

g=0;

h=0;

gg=0

%to mark where if I didn't find a good match with the SLM method

noSLM_vr=NaN(length(subjects),12);noSLM_vl=NaN(length(subjects),12);noSLM_dr=NaN(length(subjects),12);noSLM_dl=NaN(length(subjects),12);

 for s=1:length(subjects)

     for r=1:4

        if r==1

        %ventral right – distance (y and z coordinate) – simple correlations

spatial_correlation_vr(s,1)=corr(ventral_right(:,9,s),ventral_right(:,10,s),'rows','complete'); %simple correlation y axis

        spatial_correlation_vr(s,2)=corr(ventral_right(:,9,s),ventral_right(:,2,s),'rows','complete');

%piecewise regression:

        points_x=slm_optimize(ventral_right(:,10,s),ventral_right(:,9,s));

        slm_second=slmengine(ventral_right(:,10,s),ventral_right(:,9,s),'degree','linear','knots',[points_x]);

        if or(points_x(2)-points_x(1)<3,points_x(3)-points_x(2)<3)

        points_x=NaN;

        noSLM_vr(s,1)=1;

        end

        compoenet_vr(s,1)=corr(ventral_right(find((ventral_right(:,10,s))<points_x(2)),10,s),ventral_right([find((ventral_right(:,10,s))<points_x(2))],9,s));

        compoenet_vr(s,2)=corr(ventral_right([find((ventral_right(:,10,s))>=points_x(2))],10,s),ventral_right([find((ventral_right(:,10,s))>=points_x(2))],9 ,s));

        vr_sq(s,1)=slm_second.stats.R2; % %shows the R^2^

        %computes the location of the regression inflection point

        elbow_loc_vr(s,1)=points_x(2);

%y axis

        points_y=slm_optimize(ventral_right(:,2,s),ventral_right(:,9,s));

slm_second=slmengine(ventral_right(:,2,s),ventral_right(:,9,s),'degree','linear','knots',[point s_y]);

        if or(points_y(2)-points_y(1)<3,points_y(3)-points_y(2)<3)

            points_y=NaN;

            noSLM_vr(s,2)=1;

        end

        points_y_group(s,1:2)=points_y(1:2);

compoenet_vr(s,3)=corr(ventral_right([find((ventral_right(:,2,s))<points_y(2))],2,s),ventral_right([find((ventral_right(:,2,s))<points_y(2))],9,s));

compoenet_vr(s,4)=corr(ventral_right([find((ventral_right(:,2,s))>=points_y(2))],2,s),ventral_right([find((ventral_right(:,2,s))>=points_y(2))],9,s));

        vr_sq(s,2)=slm_second.stats.R2;

elbow_sensitivity_vr(s,2)=mean(ventral_right(find(ventral_right(:,2,s)<points_y(2)+4 & ventral_right(:,2,s)>points_y(2)-4 ),9,s));

        elbow_loc_vr(s,2)=points_y(2);

        elseif r==2 %ventral left

        spatial_correlation_vl(s,1)=corr(ventral_left(:,9,s),ventral_left(:,10,s),'rows','complete');

        spatial_correlation_vl(s,2)=corr(ventral_left(:,9,s),ventral_left(:,2,s),'rows','complete');

        points_x=slm_optimize(ventral_left(:,10,s),ventral_left(:,9,s));

        slm_second=slmengine(ventral_left(:,10,s),ventral_left(:,9,s),'degree','linear','knots',[points_x]);

        if or(points_x(2)-points_x(1)<3,points_x(3)-points_x(2)<3)

        noSLM_vl(s,1)=1;

        points_x=NaN;

        end

        vl_sq(s,1)=slm_second.stats.R2;

        compoenet_vl(s,1)=corr(ventral_left(find((ventral_left(:,10,s))<points_x(2)),10,s),ventral_left([find((ventral_left(:,10,s))<points_x(2))],9,s));

        compoenet_vl(s,2)=corr(ventral_left([find((ventral_left(:,10,s))>=points_x(2))],10,s),ventral_left([find((ventral_left(:,10,s))>=points_x(2))],9,s));

        elbow_loc_vl(s,1)=points_x(2);

        points_y=slm_optimize(ventral_left(:,2,s),ventral_left(:,9,s));

        slm_second=slmengine(ventral_left(:,2,s),ventral_left(:,9,s),'degree','linear','knots',[points_y]);

        if or(points_y(2)-points_y(1)<3,points_y(3)-points_y(2)<3)

            noSLM_vl(s,2)=1;

            points_y=NaN;

        end

        compoenet_vl(s,3)=corr(ventral_left([find((ventral_left(:,2,s))<points_y(2))],2,s),ventral_left([find((ventral_left(:,2,s))<points_y(2))],9,s));

        compoenet_vl(s,4)=corr(ventral_left([find((ventral_left(:,2,s))>=points_y(2))],2,s),ventral_left([find((ventral_left(:,2,s))>=points_y(2))],9,s));

        vl_sq(s,2)=slm_second.stats.R2;

        elbow_loc_vl(s,2)=points_y(2);

        elseif r==3 % dorsal right

        spatial_correlation_dr(s,1)=corr(dorsal_right(:,9,s),dorsal_right(:,10,s),'rows','complete');

        spatial_correlation_dr(s,2)=corr(dorsal_right(:,9,s),dorsal_right(:,2,s),'rows','complete');

        points_x=slm_optimize(dorsal_right(:,10,s),dorsal_right(:,9,s));

        slm_second=slmengine(dorsal_right(:,10,s),dorsal_right(:,9,s),'degree','linear','knots',[points_x]);

            if or(points_x(2)-points_x(1)<3,points_x(3)-points_x(2)<3)

            noSLM_dr(s,1)=1;

            points_x=NaN;

            end

        compoenet_dr(s,1)=corr(dorsal_right(find((dorsal_right(:,10,s))<points_x(2)),10,s),dorsal_right([find((dorsal_right(:,10,s))<points_x(2))],9,s));

        compoenet_dr(s,2)=corr(dorsal_right([find((dorsal_right(:,10,s))>=points_x(2))],10,s),dorsal_right([find((dorsal_right(:,10,s))>=points_x(2))],9,s));

        elbow_loc_dr(s,1)=points_x(2);

        dr_sq(s,1)=slm_second.stats.R2;

        points_y=slm_optimize(dorsal_right(:,2,s),dorsal_right(:,9,s));

        slm_second=slmengine(dorsal_right(:,2,s),dorsal_right(:,9,s),'degree','linear','knots',[points_y]);

        if or(points_y(2)-points_y(1)<3,points_y(3)-points_y(2)<3)

            noSLM_dr(s,2)=1;

            points_y=NaN;

        end

        points_y_group(s,5:6)=points_y(1:2);

        compoenet_dr(s,3)=corr(dorsal_right([find((dorsal_right(:,2,s))<points_y(2))],2,s),dorsal_right([find((dorsal_right(:,2,s))<points_y(2))],9,s));

        compoenet_dr(s,4)=corr(dorsal_right([find((dorsal_right(:,2,s))>=points_y(2))],2,s),dorsal_right([find((dorsal_right(:,2,s))>=points_y(2))],9,s));

        dr_sq(s,2)=slm_second.stats.R2;

        elbow_loc_dr(s,2)=points_y(2);

       % dorsal left

        else

        spatial_correlation_dl(s,1)=corr(dorsal_left(:,9,s),dorsal_left(:,10,s),'rows','complete');

        spatial_correlation_dl(s,2)=corr(dorsal_left(:,9,s),dorsal_left(:,2,s),'rows','complete');

        points_x=slm_optimize(dorsal_left(:,10,s),dorsal_left(:,9,s));

        slm_second=slmengine(dorsal_left(:,10,s),dorsal_left(:,9,s),'degree','linear','knots',[points_x]);

        if or(points_x(2)-points_x(1)<3,points_x(3)-points_x(2)<3)

            noSLM_dl(s,1)=1;

            points_x=NaN;

        end

        compoenet_dl(s,1)=corr(dorsal_left(find((dorsal_left(:,10,s))<points_x(2)),10,s),dorsal_left([find((dorsal_left(:,10,s))<points_x(2))],9,s));

        compoenet_dl(s,2)=corr(dorsal_left([find((dorsal_left(:,10,s))>=points_x(2))],10,s),dorsal_left([find((dorsal_left(:,10,s))>=points_x(2))],9,s));

        dl_sq(s,1)=slm_second.stats.R2;

        elbow_loc_dl(s,1)=points_x(2);

        points_y=slm_optimize(dorsal_left(:,2,s),dorsal_left(:,9,s));

        slm_second=slmengine(dorsal_left(:,2,s),dorsal_left(:,9,s),'degree','linear','knots',[points_y]);

        if or(points_y(2)-points_y(1)<3,points_y(3)-points_y(2)<3) % if there are not two component

            noSLM_dl(s,2)=1;

            points_y=NaN;

        end

        compoenet_dl(s,3)=corr(dorsal_left([find((dorsal_left(:,2,s))<points_y(2))],2,s),dorsal_left([find((dorsal_left(:,2,s))<points_y(2))],9,s));

        compoenet_dl(s,4)=corr(dorsal_left([find((dorsal_left(:,2,s))>=points_y(2))],2,s),dorsal_left([find((dorsal_left(:,2,s))>=points_y(2))],9,s));

        dl_sq(s,2)=slm_second.stats.R2;

        elbow_loc_dl(s,2)=points_y(2);

        end

    end

 g=g+3;

 s

 gg=gg+3;

 end

 %fisher transformation

compoenet_dl(:)=0.5*log((1+compoenet_dl(:,:))./(1-compoenet_dl(:,:)));

compoenet_dr(:)=0.5*log((1+compoenet_dr(:,:))./(1-compoenet_dr(:,:)));

compoenet_vr(:)=0.5*log((1+compoenet_vr(:,:))./(1-compoenet_vr(:,:)));

compoenet_vl(:)=0.5*log((1+compoenet_vl(:,:))./(1-compoenet_vl(:,:)));

spatial_correlation_dl(:,:)=0.5*log((1+spatial_correlation_dl(:,:))./(1-spatial_correlation_dl(:,:)));

spatial_correlation_dr(:,:)=0.5*log((1+spatial_correlation_dr(:,:))./(1-spatial_correlation_dr(:,:)));

spatial_correlation_vl(:,:)=0.5*log((1+spatial_correlation_vl(:,:))./(1-spatial_correlation_vl(:,:)));

spatial_correlation_vr(:,:)=0.5*log((1+spatial_correlation_vr(:,:))./(1-spatial_correlation_vr(:,:)));

%%

%count number of shape selective voxels in each pathway

for s = 1:length(subjects)

    num_of_vox(s,1)= sum(ventral_right(:,9,s)>0)

    num_of_vox(s,2)= sum(ventral_left(:,9,s)>0)

    num_of_vox(s,3)= sum(dorsal_right(:,9,s)>0)

    num_of_vox(s,4)= sum(dorsal_left(:,9,s)>0)

    avg_slope(s,1)=nanmean(ventral_right(ventral_right(:,9,s)>0,9,s))

    avg_slope(s,2)=nanmean(ventral_left(ventral_left(:,9,s)>0,9,s))

    avg_slope(s,3)=nanmean(dorsal_right(dorsal_right(:,9,s)>0,9,s))

    avg_slope(s,4)=nanmean(dorsal_left(dorsal_left(:,9,s)>0,9,s))

end

% slm_optimize function looks for the highest R^2^ value and determines the inflection point of the piecewise regression

function [knot2]=slm_optimize (x,y)

        clear a

        a=1;

        clear slmtry

        for i=min(x)+1:max(x)-1

        slm_second=slmengine(x,y,'degree','linear','knots',[min(x),i,max(x)]);

        slmtry(a,:)=[i,slm_second.stats.R2] ;

        a=a+1;

        end

        knot2=[min(x),slmtry(find(slmtry(:,2)==max(slmtry(:,2))),1),max(x)];

       % r2=slmtry(find(slmtry(:,2)==max(slmtry(:,2))),2);

end
