# Supplementary material for: The large-scale organization of shape processing in the ventral and dorsal pathways
Source: eLife. 2017 Oct 5;6:e27576. doi: 10.7554/eLife.27576 (PMC5659821; doi:10.7554/eLife.27576)
Supplement: Transparent reporting form [file elife-27576-transrepform.pdf]

eLIFE
1st Floor
24 Hills Road
Cambridge CB2 1JP, UK
P 01223 855340
W elifesciences.org
T @elife

eLife's transparent reporting form

We encourage authors to provide detailed information *within their submission* to facilitate the interpretation and replication of experiments. If you have any questions, please contact us: editorial@elifesciences.org.

Sample-size estimation

- You should state whether an appropriate sample size was computed when the study was being designed
- You should state the statistical method of sample size computation and any required assumptions
- If no explicit power analysis was used, you should describe how you decided what sample (replicate) size (number) to use

Please outline where this information can be found within the submission (e.g., page numbers or figure legends), or explain why this information doesn't apply to your submission:

We based our sample size on previous publications that utilized a parametric scrambling manipulation (Lerner et al., 2001). In this previous study, only nine-subjects were scanned. Since we applied a voxel-based analysis we slightly increased the number of participants (n=11, in both experiments 1 and 2) to ensure that we had sufficient power for this alternative, novel form of analysis. (data from two additional subjects were excluded because of motion artifact).

Replicates

- You should report how often each experiment was performed
- You should include a definition of biological versus technical replication
- The data obtained should be provided and sufficient information should be provided to indicate the number of independent biological and/or technical replicates
- If you encountered any outliers, you should describe how these were handled
- Criteria for exclusion/inclusion of data should be clearly stated
- High-throughput sequence data should be uploaded before submission, with a private link for reviewers provided (these are available from both GEO and ArrayExpress)

Please outline where this information can be found within the submission (e.g., page numbers or figure legends), or explain why this information doesn't apply to your submission:

- One of the aims of Experiment 2 was to replicate the findings of Experiment 1 (a biological replication) (p. 9-11;14-17)
- For each participant, eight fMRI runs were employed, to increase the reliability of the results. (p. 27-28)
- All the statistical tests were designed to show that all the effects are reliable across participants.
- Two additional participants were excluded (not analyzed), because they moved (greater than 3mm) during multiple scans. (p. 27-28)
- Exclusion: left-handed participants given the uncertainty of hemispheric organization in left-handed individuals.

Statistical reporting

- Statistical analysis methods should be described and justified
- Raw data should be presented in figures whenever informative to do so (typically when N per group is less than 10)
- For each experiment, you should identify the statistical tests used, exact values of N, definitions of center, methods of multiple test correction, and dispersion and precision measures (e.g., mean, median, SD, SEM, confidence intervals; and, for the major substantive results, a measure of effect size (e.g., Pearson's r, Cohen's d)
- Report exact p-values wherever possible alongside the summary statistics and 95% confidence intervals. These should be reported for all key questions and not only when the p-value is less than 0.05.

Please outline where this information can be found within the submission (e.g., page numbers or figure legends), or explain why this information doesn't apply to your submission:

The statistical reporting is in-line with the guidelines described above (results section p. 5-18 and methods section p. 28-34). All tests are justified as well and all values from statistical analyses reported in full.

(For large datasets, or papers with a very large number of statistical tests, you may upload a single table file with tests, Ns, etc., with reference to page numbers in the manuscript.)

Additional data files ("source data")

- We encourage you to upload relevant additional data files, such as numerical data that are represented as a graph in a figure, or as a summary table
- Where provided, these should be in the most useful format, and they can be uploaded as "Source data" files linked to a main figure or table
- Include model definition files including the full list of parameters used
- Include code used for data analysis (e.g., R, MatLab)
- Avoid stating that data files are "available upon request"

eLIFE
1st Floor
24 Hills Road
Cambridge CB2 1JP, UK
P 01223 855340
W elifesciences.org
T @elife

Please indicate the figures or tables for which source data files have been provided:

The source data for the regression analysis, the ROI analysis and the behavioral session for Experiment 1 and 2, are included in the full submission. MatLab code available upon reasonable request. fMRI raw Data was uploaded to the Carnegie Mellon University repository and available at : https://doi.org/10.1184/R1/c.3889873.v1

eLife Sciences Publications, Ltd is a limited liability non-profit non-stock corporation incorporated in the State of Delaware, USA, with company number 5030732, and is registered in the UK with company number FC030576 and branch number BR015634 at the address 1st Floor, 24 Hills Road, Cambridge CB2 1JP | August 2014
